# Design and manufacture of a low-cost 3D-printed laboratory device to measure the hyperelastic properties of polymeric films with small form factor suitable for medical devices

**DOI:** 10.1016/j.ohx.2024.e00608

**Published:** 2024-11-22

**Authors:** Hemanta Dulal, Seyedhamidreza Alaie

**Affiliations:** New Mexico State University, Las Cruces, USA

**Keywords:** Open-source testing system, 3D printing, Hyperelastic, Mooney–Rivlin, Tensile testing

## Abstract

Hyperelastic materials are extensively incorporated in medical implants and microelectromechanical systems due to their large, elastic, recoverable strains. However, their mechanical properties are sensitive to processing parameters that may lead to inconsistent characterization. Various test setups have been employed for characterizing hyperelastic materials; however, they are often costly. Recent advancements in additive manufacturing and open-source software/hardware suggest the possibility of simpler solutions in research settings for characterizing them; raising the question of whether one can characterize these materials with low-cost tools and tests that take advantage of soft and small form-factor samples. Here, the authors investigate the potential of an open-source, 3D-printed test system designed for characterizing such materials. This system is tailored for small form-factor samples (sub-mm thickness) and large elastic deformations, common in polymeric parts of minimally invasive implants. The authors developed parts using additive manufacturing for uniaxial and planar tension testing, with a low-cost image correlation method adapted for measuring large strains. Polydimethylsiloxane was chosen for demonstration of a two-parameter Mooney–Rivlin model, due to its documentation and use in biocompatible devices. The estimated Young’s and shear moduli were repeatable and consistent with the literature. Curve-fitting was challenging and dependent on the optimization choices, when data points were limited, consistent with prior reports. However, with a large number of data points and ideal optimization error choice, $C_{1}$ and $C_{2}$ were found to be close to those reported previously. This work demonstrates a low-cost, 3D-printed, open-source test setup for characterizing hyperelastic materials using a two-parameter Mooney–Rivlin model with reasonable accuracy.

## Graphical abstract

**Figure:**
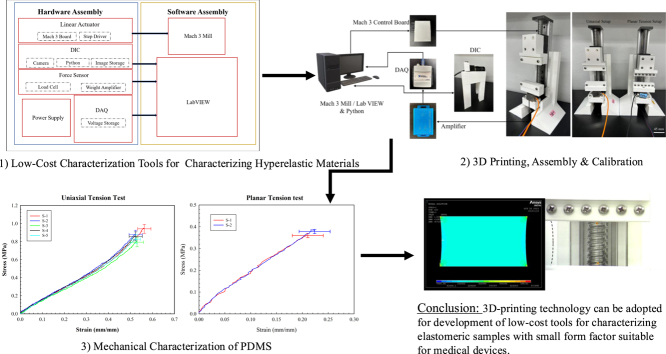

**Table:** 

**Specifications table**	
Hardware name	Hyperelastic Material Test Setup (HypeMaTS)
Subject area	• Engineering and material science
Hardware type	• Other: Material Testing
Closest commercial analog	• Mark-10: FS05 series • MTS: Criterion series 40
Open source license	CC-BY-4.0
Cost of hardware	1471.16
Source file repository	https://osf.io/jg7uv/files/osfstorage
Curve Fitting Code	https://github.com/seyeala/HX_Mooney_Rivlin-CurevFit.git

## 1 Hardware in context

Research involving hyperelastic materials has been growing, particularly in the fields of medical devices [1], [2] and microsystems [3], [4], and in various applications simulating their mechanical behavior is critical. These simulations often require complex models that necessitate careful curve fitting [5], [6]; also, their fabrication involves numerous process parameters. Given the variability in various parameters, relying solely on data from the literature can be challenging. Although well-established characterization methods and tools are available [7], [8], they are often prohibitively expensive for most laboratory environments. The combination of high characterization costs, model complexity, and sensitivity to the process parameters highlight the need for a low-cost in-house solution for characterizing hyperelastic materials. We propose that a low-cost open-source system, that utilize recent advancements in open-hardware and software if tailored to the dimensions relevant to medical devices and microsystems, could be utilized for characterization of hyperelastic properties with a level of accuracy sufficient for laboratory use. To test this hypothesis we develop a test setup using 3D printed parts, and open-source software solutions. We validate this system by characterizing a hyperelastic material and model that are widely used in the literature.

A low-cost characterization system can be impactful in multiple research areas. Hyperelastic materials are common in medical implants due to their unique properties. Some of the elastomers commonly incorporated in biomedical and micromechanical systems are polydimethylsiloxane (PDMS), silicone rubber, and thermoplastic polyurethane (TPU). These elastomers are optically transparent, chemically inert, with a simple fabrication process [9]. These features are advantageous in microfluidic device [3]. Among various applications for hyperelastic materials, Micro electro-mechanical systems [10] (MEMS) employ PDMS as a substrate material due to its simple fabrication process and ability to take the shape of micro-patterns during the soft lithography [3], [4] process. For instance, micropumps [11] and microvalves are made from PDMS. Lately, considerable interest in employing PDMS-based elastomers for the fabrication of mitral valves [1], soft occluders [2] from the polyurethane (PU) is evident in the area of medical devices. Due to the flexibility of these materials, they are used in drug delivery [12], wearable stretchable electronics [13], cell trapping [14] and biological force measurements, demonstrating various applications.

Simpler mechanical property tests, use of open-source materials, and tailoring tests for samples and the research needs have been of interest recently. A comprehensive characterization of mechanical property may include uniaxial tensile, biaxial test, and planar tension tests that is commercially available. However the tests are typically costly since they are typically equipped to characterize a wide range of samples [7], [8]. Moreover, they are not tailored to take advantage of polymeric samples to reduce the complexity and cost of the systems. Recent studies leveraged samples’ small dimensions, materials, and open-source resources, and introduced cost-effective approaches for characterization of the mechanical/physical properties. At the bulk scale a low cost universal tensile test machine was introduced [15], however with 900 kg load-cell and all metal parts, the design was not open-source and suitable for characterizing hyperelastic materials. Studies on microscale metal samples employed miniaturized testing setup [16] have focused on developing open-source, custom-made low-cost mechanical characterization test setups. An attempt to characterize hyperelastic material using open-source radial stretching [17] system was developed to substitute uniaxial and biaxial tests. These reports demonstrate the need for a cost-effective, open-source test setup, that is tailored for small-scale elastomeric samples, for the characterization of hyperelastic material.

Open-source hardware, enabled by additive manufacturing, and open-source software are other novel resources that can be utilized in developing customized tools for characterization of hyperelastic materials. Recently, the additive manufacturing technique has caught much attention due to low-cost [18], [19] and flexibility in fabricating complex geometry [20]. Given the advancement in 3D printing technologies, fabricating test setups using 3D printed parts is a growing research interest. Fabrication of particle image velocimetry (PIV) setup using 3D printed parts [21] demonstrated its capability to be an ideal alternative for low-cost fabrication techniques. In another instance, the development of open-source opto-mechanical components from 3D printing [22] demonstrated similar results in comparison to its industrial counterparts. To our best knowledge, the development of a 3D-printed mechanical test setup for characterizing hyperelastic material has been less explored. This gap has served as motivation for the development of an open-source, 3D printed test setup that is tailored for small form-factor hyperelastic materials that are typically used in medical implants/devices. Typically, open-source testing setup in a typical strain-controlled uniaxial, pure shear and inflation test [23], embrace digital image correlation technique [24], [25] (DIC) to measure strain [26]. In this technique, special patterns (speckles) are sprayed in the testing sample, and strain is measured based on the change in position of these special marks. Open-source digital correlation (DIC) algorithms such as NCorr [27] is widely adopted to track particles and require a large area of sample covered with speckle. However, restriction in speckle’s size and its distribution over the test sample area affect the result & mechanical properties [28] of the material under study. Thus, the DIC technique, that uses the fewest possible sets of speckles without changing the stiffness of the material under study and yields correct strain results, is advantageous, similar to commercial optical extensometers. It is notable however, use of low cost parts and open-source software, while reduce the cost, may also reduce the accuracy of the measurement. For instance, a low-cost camera without magnification can be two orders of magnitude less accurate than the positional accuracy of a linear actuator. These evident drawbacks, despite the potentials, raise the question whether such a system can be utilized for effective characterization of hyperelastic materials. In order to understand the potential, in this work, 3D printed parts in conjunction with open-source software is adopted for the development of test setups capable of providing reasonably precise characterization tools for small form factor hyperelastic samples.

Various complex constitutive models have been employed to simulate mechanical behavior rubbers since most applications involve large elastic deformations. Applications such as thin film sensors, accelerators, and flexible adaptable fluidic lenses demonstrated large deformations [29], [30], [31]. Studies on mechanical behavior [29], [32], [33], [34] of commercially available elastomers, such as PDMS and TPU, demonstrated non-linear [34], [35] behavior. This non-linear behavior is well documented [29], [36], [37] and can be attributed to process parameters such as cross-link density [33], [38], [39] thickness [32] and curing temperature [34] respectively. Recently, efforts to characterize stress versus strain behavior of PDMS [40], [41], [42], [43], [44] and polyurethane [38], [39], [45] led researchers to follow the hyperelastic material model. Research efforts to provide metrics on hyperelasticity based upon observed phenomenological behavior and mechanistic approach resulted in Ogden [46], Mooney–Rivlin [47], [48], Yeoh [49], Neo-Hookean [50] and Arruda–Boyce [51] material models. These models rely on invariants from Cauchy–Green deformation tensors (Mooney–Rivlin, Neo-Hookean) and principal stretches (Ogden).

Among these models, the Mooney–Rivlin model provides more accurate results [44] for large strains when compared to other models. Mooney–Rivlin model is a special case of the polynomial model where its order (n = 1, 2, 3) results in 2, 3, 5, and 9 parameter models respectively. Most of the studies [10], [52], [53], focused on the characterization of elastomers (PDMS, PU), involve incompressible two-parameter Mooney–Rivlin model (Eq. (1)), which is well founded for up to 100% of strain. The invariant-based model involves more than one hyperelastic material constant such as ‘$C_{10}$’ and ‘$C_{01}$’, which are commonly referred as ‘$C_{1}$’ and ‘$C_{2}$’ respectively:

(1) $$W=C_{10}\left(I_{1}-3\right)+C_{01}\left(I_{2}-3\right)$$

where, *W* is the Strain-Energy Density

(2) $$I_{1}=\lambda_{1}^{2}+\lambda_{2}^{2}+\lambda_{3}^{2}$$

(3) $$I_{2}=\lambda_{1}^{2}\cdot \lambda_{2}^{2}+\lambda_{2}^{2}\cdot \lambda_{3}^{2}+\lambda_{3}^{2}\cdot \lambda_{1}^{2}$$

the shear modulus is given by

(4) $$\mu =2\left(C_{01}+C_{10}\right)$$

and Poisson’s ratio is computed as:

(5) $$\nu =\sqrt{1-\frac{E_{uniaxial}}{E_{planar}}}$$

Characterization of these hyperelastic coefficients, in contrast with elastic materials, typically involves multiple types of tests, complex models, and sometimes inconstancy of the coefficients reported in the literature. Estimation of these coefficients involves a combination of experimental tests such as uniaxial tension, uniaxial compression, biaxial tensile tests, pure shear, planar tension, and/or bulge test [54] as these coefficients are associated with a change in volume and shape of a sample. In another instance, estimation of hyperelastic properties for filled elastomer was carried out using uniaxial, pure shear, and inflation test [23]. However, the results for $C_{1}$ and $C_{2}$ from some studies are inconsistent with each other, which demonstrates the challenge in engineering design and fabrication of PDMS devices. Various works reported different values for hyperelastic material constants for PDMS, such as those obtained from bulge test [10], $C_{1}$ = 75.5 kPa and $C_{2}$ = 5.7 kPa, and those obtained from uniaxial tension test [53], $C_{1}$ = 34.3 kPa and $C_{2}$ = 46.9 kPa. Estimation of material coefficient based upon uniaxial tension test only leads to less accurate results [6], [55], [56]. Furthermore, an analytical approach in conjunction with biaxially inflating flat disk experiment [52] was adopted to evaluate hyperelastic material coefficient ($C_{1}\&C_{2}$) for PDMS, and resulted in higher values where $C_{1}$ = 270 kPa and $C_{2}$ = 10.8 kPa. A similar inconsistent trend is observed in the case of polyurethane as reported values from [38], [39] are far from each other. Given that the mechanical properties of PDMS depend significantly on process parameters, it is likely that inconsistencies are rooted in process control or simply non-uniqueness of these coefficients. The large number of process parameters, test settings, and sometimes inconsistency highlights the importance of in-house estimation, characterization, and control of material properties, especially in a research setting that requires consistent mechanical properties for PDMS.

Another characterization challenge is the fact that finding optimal hyperelastic parameters is mathematically non-unique under limited loading conditions (an inverse problem [6]), which is common in nonlinear elasticity. This fact contributes to the difficulties in curve-fitting the correct parameters that are optimal across all load conditions, rather than just a few. This challenge in curve fitting hyperelastic parameters has been reported in various works, highlighting that the curve fitting can be delicate [5], [6] and the curve-fit parameter can be highly sensitive to data. Evidently, various choices for optimizing error could be adapted such as absolute error, mean square error, or percentage error in stresses. While one may assume that the difference between these assumption for curve fitting is insignificant, the literature suggests otherwise [6], [57], [58]. Various work suggested that optimizing the error in’ $\sigma /\left(\lambda^{2}-\frac{1}{\lambda }\right)$’ rather ‘σ’, is less sensitive to the data and is more advantageous. Also other work highlighted the need for meticulous/uniform sampling from the stretches in various regions. These reports indicate the need for a careful curve fitting, choice of optimization technique, and number/types of tests, which in turn further highlight the need for a low-cost in-house characterization in a research laboratory.

**Fig. 1:**
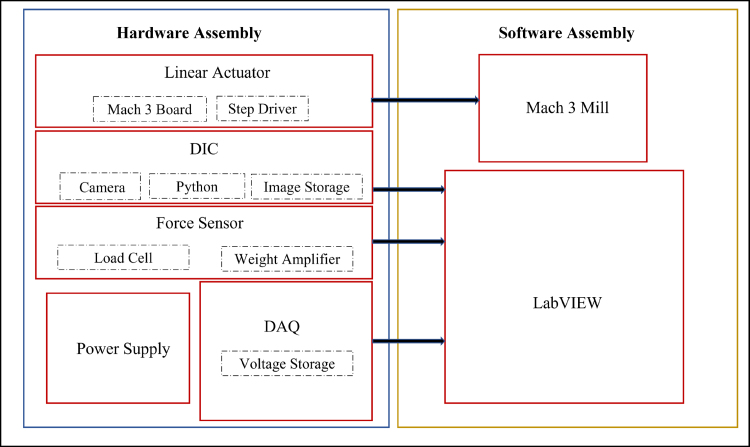
Block diagram demonstrates the communication between hardware and software modules.

In this work, we report a process flow that demonstrates the development of a novel bench top, open-source, low-cost 3D printed testing system for mechanical characterization of hyperelastic material (Fig. 1). We hypothesize that open-source uniaxial and planar tensile test setups, developed using fused deposition modeling (FDM) parts, can provide reasonable results for the characterization of hyperelastic materials, with sub-mm thick sample size. To test our hypothesis, we choose PDMS (Sylgard 184) as a hyperelastic material to be characterized, due to its wide applications in medical devices, and the availability of its process and characterization data in the public domain. Here, we study the stress–strain behavior of Sylgard 184 (10:1 ratio) cured at 100 °C using the open-source DIC technique with a two-point speckle pattern. Subsequently, we report a route to estimate consistent/repeatable two-parameter Mooney–Rivlin coefficient using commercially available software. Furthermore, material constants obtained from curve fitting uniaxial & planar tension test data will be used in a finite element analysis for validating the experiment.

## 2 Hardware description

**Fig. 2:**
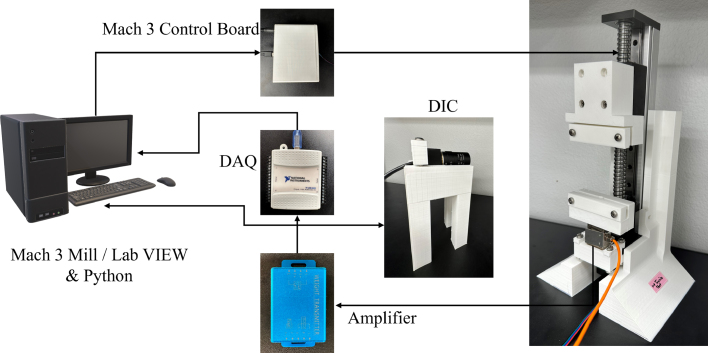
Test setup workflow diagram.

The design is inspired single-column structure with a larger base area for improved stability. Uniaxial and planar test tools, see Fig. 2, consists of the Base, Linear Actuator, Grippers set, Force Sensor, strain gauge amplifier, and data acquisition modules, see Fig. 1. An aberration-correct camera is used to measure the stretch in a sample under test. Prior studies [59] on the development of a small uniaxial testing machine suggested that a lead screw-based mechanism is well suited for a miniature testing setup. Therefore, here a lead screw-based linear actuator (Befenybay, SFU1605), with a 150 mm travel length, is employed for applying strains. The movement and speed of the actuator are controlled by a 5-axis Mach3 USB controller board (Figs. 1, 2) powered by a 24 V power supply and Mach3 controller graphics user interface (GUI). In addition, a micro-step drive board is used for driving the stepper motor and controlling the micro-steps according to the peak current, current, and pulse/revolution requirement.

The use of 3D-printed structural components results in a more compliant tool. Additionally, the linear actuator is less precise than the industry-standard ones typically used in tensile tests. This setting allows for a cost-effective setup at the expense of a lower strain measurement accuracy. To compensate for this shortcoming, the stretch in the sample is directly measured using a camera, whose operation is independent of the structural compliance of the 3D-printed parts or the precision of the actuator. Moreover, the stretch of elastomeric samples with a small form factor requires small force, which also mitigates the adverse effects of compliant 3D-printed components. This setup is particularly suitable for scenarios, where the characterization of small form factor elastomeric samples is required, with significant deformation and moderate accuracy. Such a configuration is ideal for laboratories that work with elastomers and aim to characterize the relationship between their mechanical properties and processing parameters.

### 2.1 Force sensing unit

Force sensor setup consists of one S-Type load cell (CALT, DYLY-106) with a maximum weight capacity of 1 kg (Uniaxial), 5 kg (Planar), and a 4–20 mA rated strain gauge amplifier(DY510). Load cells with lower capacity, such as 100 gr, could be used for improved accuracy based on the expected maximum force (that depends on the sample and its thickness), which may require some adjustment in the design of the Load cell Bracket and Mount. The amplified voltage from the strain gauge amplifieris recorded using a 14-bit data acquisition system (NI-6009, National Instruments). The force sensing unit consists of a 3D printed bracket (C1002) secured on the linear actuator using a bracket and M4 Hex Nut/Bolt combination. The load cell (DYLY 105) is secured firmly on the bracket using an M6 screw. The gripper base (UNI1002) is attached and fixed on top of the load cell using M4 screw. Upon securing the load cell, it is electrically connected to the strain gauge amplifier. The amplifier has a voltage output showing the force which is connected to a DAQ. The DAQ is connected to a computer through a USB 3.0 cable and is run using LabVIEW Code. The strain gauge amplifier and DAQ system are powered by a 12 V DC power supply.

### 2.2 Sample grip mechanism

The sample gripping setup consists of two 3D-printed pairs of clamps that are named bottom (UNI1002) and top (UNI1001) grippers. Two distinct designs are adopted for the bottom and top grippers as the bottom gripper is attached to the load cell using M4 and the other is attached to the linear actuator bed (top). The bottom grippers consist of two holes to incorporate metric hex M4 nut which is then secured with the aid of adhesives. Top gripper geometry is simple and identical in size and shape to ensure uniformity in clamping.We acknowledge that the surface roughness of 3D-printed grippers is high and leads to uneven grip. However, in the case of rubber samples such PDMS (which are the focus of this work), the effect of surface roughness can be minimal, if it is gripped firmly. Modification of the grippers by inclusion of metal plates is a possibility for other types of samples. Each gripper has a bar to be tightened on the top of the gripper using bolts/screws. One of the major differences between uniaxial and planar grippers is the number of nuts/bolts assemblies employed for gripping samples. The planar test has 16 gripping points altogether, which prevent sample/films from horizontal motion, whereas the uniaxial test design includes 4 gripping points. This difference allows for testing samples/films that are very narrow or wide.

### 2.3 Digital image correlation

The sample images during the uniaxial test are captured using a camera (ELP-USBFHD01M-SFV). The camera employed in this setup is aberration-corrected for computer vision applications. OpenCV package was used to detect the two red dot marks, on the sample, from the image, which correspond to the sample’s stretch. A wide range of adjusting parameters such as brightness, color saturation, and view angle is available for this camera. It is also compatible with USB 2.0 protocol in various operating systems. The camera stand is 3D printed using an FDM printer (Anycubics) with PLA material at 100% infill. The camera is secured in the stand (see Fig. 2) using M4 screws and connected to the computer with a USB 2.0 cable.

### 2.4 Graphic user interface assembly

The operation of the tensile test requires two separate software modules (Fig. 1) namely: (1) Mach3 Mill, and (2) LabVIEW which are operated independently and are synchronized manually. The actuator’s motion is controlled by the Mach 3 Mill graphic user interface through the Mach 3 USB control board, which controls the steps per unit movement (Fig. 5). Users can specify the maximum travel distance and the velocity manually, as well as using simple g-code scripts to control the movement with an accuracy of ± 0.03 mm in the Mach 3 mill module.

The LabVIEW module includes a loop with a customizable delay time, allowing the user to select both the frequency and duration of force and displacement sampling. Before entering the loop, initial inputs are read, and a Python program is initiated. Inside the loop, LabVIEW executes three consecutive frames. Each frame begins only after all operations in the previous frame are completed. Once the third frame finishes, the sequence repeats. This structure ensures precise control over the timing of the readings. In the first frame, a Python program calculates the distance between two dots on the sample (in pixels) and their positions. Simultaneously, a DAQ Assistant measures the voltage output from all DAQ channels (including the one connected to the load cell). The Python program, using OpenCV, detects two circles by masking their colors and calculates the pixel distance between their centers, returning a vector of the pixel distances and positions. After the Python function returns its output, the DAQ Assistant immediately samples the voltage 50 times at a 10 kHz frequency, which returns a matrix of voltage values with 50 rows. In the second frame, the rows of the voltage matrix are averaged, and the matrix is reduced to a single vector. This vector is concatenated with the pixel position vector, then forms one vector for each instance of complete measurement. The vector is then added as a new row to a previously stored CSV file, which represents a new measurement or datapoint. In the third frame, a delay time (typically from 2 to 5 s) is implemented. This delay ensures that the time between voltage and strain measurements is negligible in comparison with the time between instances of complete measurements. The exact delay time depends on the speed of the computer and its operating system, and can be adjusted by trial and error. However, a delay, ranging from 2 to 5 s, is typically sufficient. Note: If a slow overall sampling rate is chosen, the speed of the Mach3 mill should be reduced to ensure enough data points are collected. If faster measurements (< 1 s per measurement) are required, in the event that the viscoelastic properties matter, careful attention to timing is necessary. Reducing the number of samples in the DAQ assistant reduces the lag between strain/stress measurements, which enables faster overall measurement. After all iterations are complete, the Python session and program are closed.

### 2.5 Key aspects of the system

• The system is tailored for characterization of elastomers with a small thickness.
• The system has large displacement range to test samples under uniaxial & planar tension configuration (Strain > 40%).
• Load cell capable to measure small forces for thin-film elastomeric samples.
• High resolution computer vision camera and DIC method for non-contact strain measurement.
• Automatic with user-defined system control parameters (Velocity, acceleration, travel distance).
• 3D printed light material advantageous for portable setup.
• Cost is under $ 1500 remarkably cheaper than commercial uniaxial and planar tension test combined.

***Design files***

## 3 Design files summary

**Table:** 

Design filename	File type	Open source license	Location of the file (reserved)

Base Bottom	CAD	CC-BY-4.0	https://osf.io/jg7uv/files/osfstorage
Load cell Bracket	CAD	CC-BY-4.0	https://osf.io/jg7uv/files/osfstorage
Load cell Mount	CAD	CC-BY-4.0	https://osf.io/jg7uv/files/osfstorage
Uniaxial Test Top Gripper	CAD	CC-BY-4.0	https://osf.io/jg7uv/files/osfstorage
Uniaxial Test Bottom Gripper	CAD	CC-BY-4.0	https://osf.io/jg7uv/files/osfstorage
Planar Test Top Gripper	CAD	CC-BY-4.0	https://osf.io/jg7uv/files/osfstorage
Planar Test Bottom Gripper	CAD	CC-BY-4.0	https://osf.io/jg7uv/files/osfstorage
Uniaxial Test Top Gripper Bar	CAD	CC-BY-4.0	https://osf.io/jg7uv/files/osfstorage
Uniaxial Test Bottom Gripper Bar	CAD	CC-BY-4.0	https://osf.io/jg7uv/files/osfstorage
Planar Test Bar Gripper	CAD	CC-BY-4.0	https://osf.io/jg7uv/files/osfstorage
Camera Bracket	CAD	CC-BY-4.0	https://osf.io/jg7uv/files/osfstorage
Camera Stand	CAD	CC-BY-4.0	https://osf.io/jg7uv/files/osfstorage
Electronic Case	CAD	CC-BY-4.0	https://osf.io/jg7uv/files/osfstorage
Electronic Case Lid	CAD	CC-BY-4.0	https://osf.io/jg7uv/files/osfstorage
Base Top	CAD	CC-BY-4.0	https://osf.io/jg7uv/files/osfstorage

***Bill of materials***

## 4 Bill of materials summary

**Table:** 

Designator	Component	Number	Cost per unit ($)	Total cost ($)	Source of materials	Material type

DIC Camera	ELP-5–50	1	108	108	https://www.amazon.com/ELP-5-50mm-Varifocal-Vari-Focus-Raspberry/dp/B0BVFKTM6Z?ref_=ast_sto_dp	Electronic Component

USB2.0 extension		1	6	6	https://www.amazon.com/AmazonBasics-Extension-Cable-Male-Female/dp/B00NH11PEY/ref=sr_1_1_ffob_sspa?crid=3OUEKPEM1NMMA&keywords=usb%2Bextension%2Bcable&qid=1694116646&sprefix=USB%2BExtension%2Caps%2C121&sr=8-1-spons&sp_csd=d2lkZ2V0TmFtZT1zcF9hdGY&th=1	Electronic Component

M4 Bolts		50	0.2944	14.72	https://www.mcmaster.com/catalog/129/3393/92000A242	Metal

M6 Bolts		25	0.49	12.27	https://www.mcmaster.com/catalog/129/3393/92000A440	Metal

M4 Nuts		100	0.0659	6.59	https://www.mcmaster.com/catalog/129/3584/91828A231	Metal

M4 Washers		100	0.0343	3.43	https://www.mcmaster.com/catalog/129/3628/98689A113	Metal

Extension Cable	GE 6	1	13	13	https://www.amazon.com/GE-Outlet-Protector-Extension-14092/dp/B00DOMYL24/ref=sr_1_34?crid=1ZVWWHWYMRVWO&keywords=extension%2Bcord&qid=1694116532&sprefix=extension%2Bcord%2Caps%2C117&sr=8-34&th=1	Electronic Component

150 mm Length Linear Stage Actuator	Befenybay SFU1605	2	78.8	157.6	https://www.amazon.com/Befenybay-Actuator-Ballscrew-SFU1605-Stepper/dp/B08DKB5G6F/ref=asc_df_B08DKB5G6F/?tag=hyprod-20&linkCode=df0&hvadid=475788732916&hvpos=&hvnetw=g&hvrand=3060184322775087062&hvpone=&hvptwo=&hvqmt=&hvdev=c&hvdvcmdl=&hvlocint=&hvlocphy=1022560&hvtargid=pla-1185450218066&psc=1	Metal

Load Cell 1 kg	DYLY-106	1	142.38	142.38	https://www.ato.com/micro-load-cell-s-type-1kg-to-50kg?affiliate=shopping&gclid=EAIaIQobChMI24mD5LnI_gIVzhJMCh0WZwKVEAYYAiABEgKwufD_BwE	Metal

Load Cell 5 kg	DYLY-106	1	45.69	45.69	https://www.ato.com/micro-load-cell-s-type-1kg-to-50kg?affiliate=shopping&gclid=EAIaIQobChMI24mD5LnI_gIVzhJMCh0WZwKVEAYYAiABEgKwufD_BwE	Metal

Strain Gauge Amplifier (Load Cell Weight Transmitter)	ATO	2	81.99	163.98	https://www.amazon.com/Transmitter-Transducer-Amplifier-Compression-Converter/dp/B08CN3NBSC/ref=asc_df_B08CN3NBSC/?tag=hyprod-20&linkCode=df0&hvadid=598708429145&hvpos=&hvnetw=g&hvrand=16299711299919447050&hvpone=&hvptwo=&hvqmt=&hvdev=c&hvdvcmdl=&hvlocint=&hvlocphy=1022560&hvtargid=pla-1813408525425&th=1	Metal

DC power supply	12 V-2 A	3	12	36	https://www.amazon.com/100-240V-Transformers-Switching-Applications-Connectors/dp/B077PW5JC3/ref=sr_1_1_sspa?crid=2ME0I9V9GZ5P3&keywords=12v+dc+power+supply+2a&qid=1694116025&sprefix=12v+dc+power+supply%2Caps%2C119&sr=8-1-spons&sp_csd=d2lkZ2V0TmFtZT1zcF9hdGY&psc=1	Electronic Component

Mach 3 stepper driver	MACH3USB	2	28.19	56.38	https://www.amazon.com/MACH3-Motion-Controller-Breakout-Engraving/dp/B07KYM15PC/ref=sr_1_3_sspa?crid=12A42GH2TX2I8&keywords=mach3+usb+5+axis+stepper+board&qid=1682547014&s=electronics&sprefix=mach3+usb+5+axis+stepper+borad%2Celectronics%2C100&sr=1-3-spons&psc=1&smid=A2K5DI8VX12AN1&spLa=ZW5jcnlwdGVkUXVhbGlmaWVyPUEyS0c3Q1lVQks3QkNZJmVuY3J5cHRlZElkPUEwMjA5MzYzM1Q3OTA2UFFIVk9ETyZlbmNyeXB0ZWRBZElkPUEwMjU3NzMyMTNSNlg5SDhLVDgyOSZ3aWRnZXROYW1lPXNwX2F0ZiZhY3Rpb249Y2xpY2tSZWRpcmVjdCZkb05vdExvZ0NsaWNrPXRydWU=	Electronic Component

Nema 17 stepper controller	TB6600	2	17.99	35.98	https://www.amazon.com/DaFuRui-Stepper-Controller-Subdivision-Upgrade/dp/B07KGQDQMN/ref=sr_1_1_sspa?keywords=Nema+17+stepper+driver&qid=1682547174&sr=8-1-spons&psc=1&spLa=ZW5jcnlwdGVkUXVhbGlmaWVyPUEzQlNHNDg2Sjg1RUdPJmVuY3J5cHRlZElkPUEwOTIzMjgzMjVCWUFGVlZYVEcwSyZlbmNyeXB0ZWRBZElkPUEwMjcyMzgwMUhRWEVSWDJWN1QxTyZ3aWRnZXROYW1lPXNwX2F0ZiZhY3Rpb249Y2xpY2tSZWRpcmVjdCZkb05vdExvZ0NsaWNrPXRydWU=	Electronic Part

PLA Filament	OVERTURE PLA	3	18.99	18.99	https://www.amazon.com/OVERTURE-Filament-Consumables-Dimensional-Accuracy/dp/B07PGZNM34/ref=asc_df_B07PGZNM34/?tag=&linkCode=df0&hvadid=343819133924&hvpos=&hvnetw=g&hvrand=11011565794907950185&hvpone=&hvptwo=&hvqmt=&hvdev=c&hvdvcmdl=&hvlocint=&hvlocphy=9030610&hvtargid=pla-757472807961&ref=&adgrpid=71843848760&th=1	PLA

NI DAQ	USB-6009	1	259.99	259.99	https://www.amazon.com/Acquisition-779026-01-National-Instruments-USB-6009/dp/B0819ZJSYL/ref=sr_1_1?crid=J980C8BUYULE&keywords=New+NI+USB-6009+Acquisition+Card+Module+Data+Multifunction+DAQ&qid=1682544881&sprefix=new+ni+usb-6009+acquisition+card+module+data+multifunction+daq%2Caps%2C403&sr=8-1	Electronic Component

Small Calibration weights	UCEC	1	17.99	17.99	https://www.amazon.com/Calibration-10mg-100g-Precision-Stainless-Jewellry/dp/B085N5NH8Q/ref=sr_1_18?crid=2SA1WKOQSZ4C3&keywords=calibration+weight&qid=1682547730&sprefix=%2Caps%2C208&sr=8-18	Stainless Steel

1 kg Calibration Weight	HFS(R) M2	1	18.99	18.99	https://www.amazon.com/HFS-Chrome-Scale-Calibration-Weight/dp/B07HF3LXWQ/ref=sr_1_3?crid=20IOCG61DP4B8&keywords=1%2Bkg%2Bcalibration%2Bweight&qid=1682547941&sprefix=1%2Bkg%2Bcalibration%2Bweight%2Caps%2C110&sr=8-3&th=1	Stainless Steel

## 5 Build instructions

### 5.1 Test stand

**Fig. 3:**
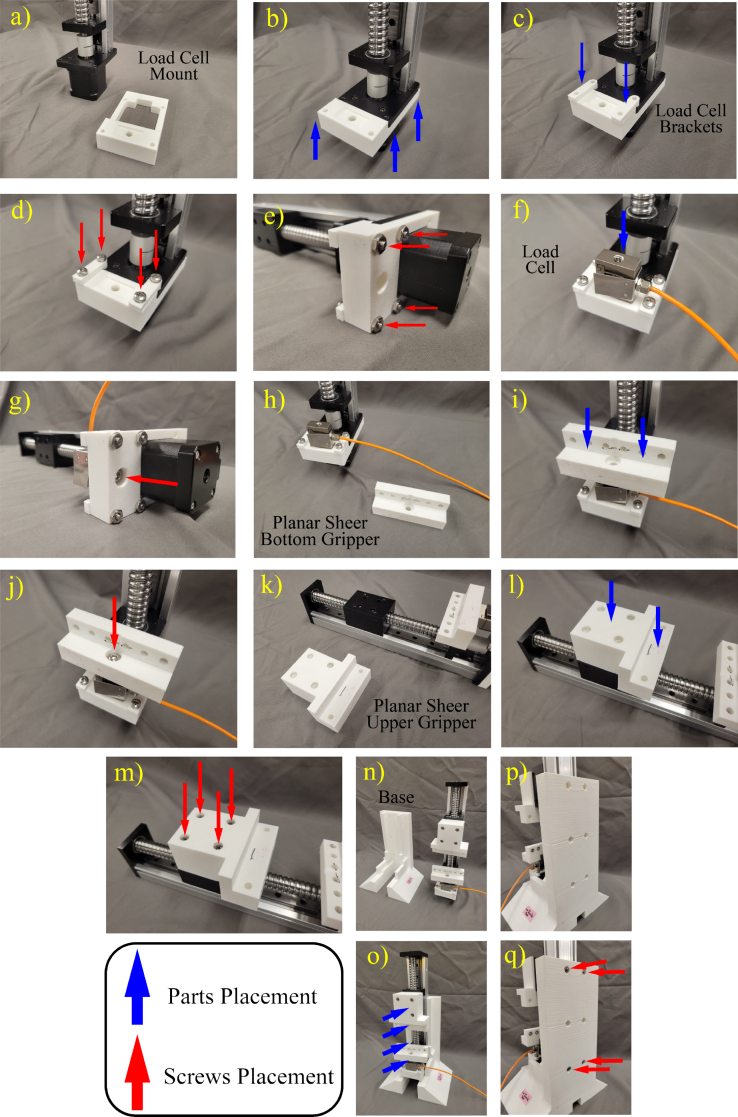
Material Assembly steps including: (a) starting with the load cell mount and the actuator, (b) mounting the load cell mount on the stepper motor, (c) mounting the brackets on the load cell mount and the stepper motor, (d) fastening the bolts, (e) fastening the nuts, (f) placing the load cell on the load cell mount, (g) attaching the load cell to the load cell mount with a bolt, (h) placing the assembled parts upright, (i) placing the bottom gripper on the load cell, (j) fastening the gripper on the load cell by a bolt, (k) placing the assembled parts on the table, (l) placing the top gripper on the moving bed, (m) fastening the top gripper on the bed, (n) placing the assembled part and the 3D printed base upright, (o) attaching the Base Bottom base to the actuator with screws.

Step by step assembly of the test stand is depicted in Fig. 3. First, a special mount (Load cell Mount; Fig. 3a, b) and brackets (Load Cell Bracket ; Fig. 3c) are attached to the stepper motor (Fig. 3d, e). Next the load cell is attached to the mount (Fig. 3f, g). Next, the bottom gripper (1D Tensile Bottom Gripper) is installed on top of the load cell using M6 screws (Fig. 3h, i, j). The top gripper (1D Tensile Top Gripper) is attached to the moving bed of the actuator using M4 bolts (Fig. 3k, l, m). Finally Base Bottom is attached to the actuator (Fig. 3n, o, p, q). Base Top could also be attached optionally, which could assist in inverting the stand for calibration (handling calibration weights from the load cell). The camera is secured on a 3D printed stand (Camera Stand; Fig. 2) using a bracket (Camera Bracket) by tightening the M4 screw. The Mach 3 control board and Stepper driver module are also secured inside a 3D printed box (Electronic Box Case) for the safety of the electronic components. All the design files are available in provided repository (Specification Table).

**Fig. 4:**
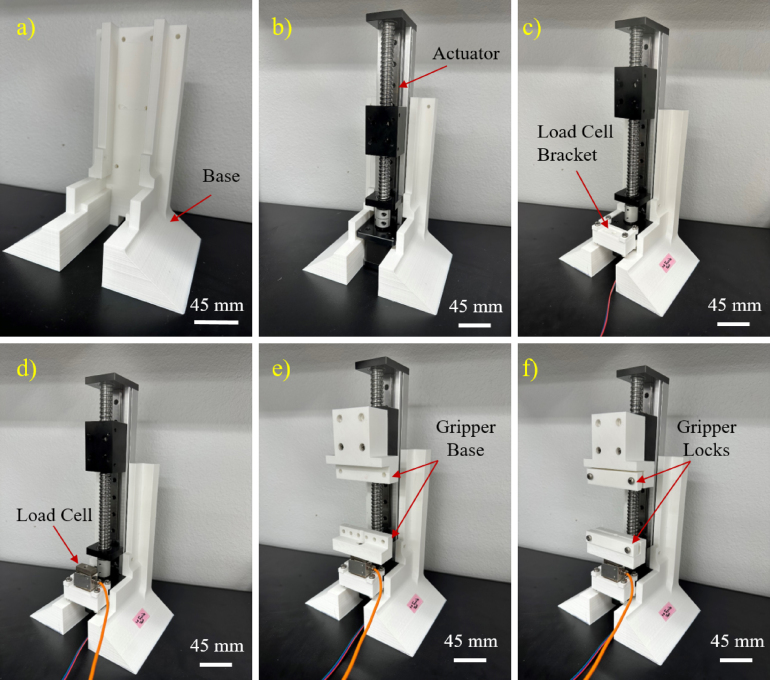
Step by-step guide for building the stand for uniaxial test assembly.

Fig. 4 further demonstrates various stages of the test stand assembly, in which parts are assembled with better clarity. The steps include the 3D printed base (Fig. 4a) that is integrated with the actuator (Fig. 4b), attaching the Load Cell Bracket (Fig. 4c), the load cell (Fig. 4d), Uniaxial Test Bottom Gripper Bar (Fig. 4e), and attaching Uniaxial Test Top Gripper Bar (Fig. 4f).

### 5.2 Electronics

**Fig. 5:**
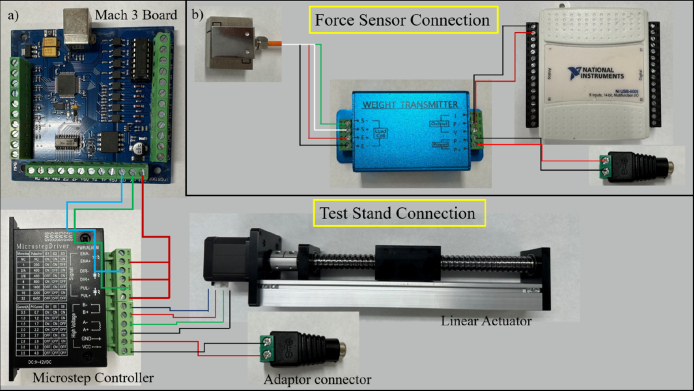
Electrical connection diagram for (a) controlling test platform (b) force sensing unit and DAQ system.

The electrical connection of the components such as the load cell and stepper motor is depicted in Fig. 5. They are powered by a 12 V DC power supply. The ＋5V terminal in the Mach 3 USB board is connected to all positive terminals (ENA+, DIR+, PUL+) of the micro-step driver signal output plug. The pulse (XP) and direction (XD) control terminal of the Mach3 board are connected to the negative terminal (DIR-, PUL-) of the micro-step driver. The motor is connected to the micro-step driver board at the high voltage output terminal plug located at the bottom as shown in Fig. 3g and Fig. 5; and is powered by a 24 V DC power supply from GND and VCC terminals. This board is connected to the Mach3 USB board that is controlled by a laptop using a standard USB 2.0 cable. The force sensing unit’s wires (Load Cell) are connected to the strain gauge amplifier terminals (S+, S-, E+, E-). The other end of the amplifier terminals (P-, V) are connected to the DAQ analog terminal (GND, A0), and the power terminals (P-, P+) are connected to a 12 V power supply (see Fig. 5).

## 6 Operation instructions

First, the Mach3 control board is connected to the computer by a USB cable, and the micro-step driver module is powered by a 24 V DC power supply (Fig. 2). The load cell and the strain gauge amplifier are connected and powered by a separate power supply (12 V). The data acquisition system and DIC camera setup are connected to the computer with a USB cable (Fig. 2). Once the hardware module is powered, Mach3Mill and LabVIEW software are opened for executing the uniaxial/planar tensile test setup with user-specified distance & velocity and capturing images simultaneously. A Gripper head velocity of 0.4 mm/s is used for uniaxial and planar tests. A sample with red dots is then mounted on the uniaxial test setup and secured using the nut/bolt combination. The sample is stretched slightly by running Mach3Mill to avoid buckling and ensure that it is straight. In order to capture high-quality images, the camera is opened to adjust the light and resolution as LabVIEW returns errors upon disturbances on the camera, while detecting the red marks. The LabVIEW virtual instrument (VI) is run for 10 cycles without running Mach3Mill to ensure that DIC recognizes red dots and corresponding voltage output, along with the images that are stored in the computer. The images captured by the VI are stored in a local drive folder. The voltage output along with pixel distance ‘Δl’ from Python is stored in an MS Excel file. Upon successful testing of the DIC camera, a simple text file “gcode.txt” was created, where the maximum travel distance and the velocity for the test were specified. The code is opened in Mach3Mill GUI using the “Load code” pane, and the coordinates are displayed in the “Program Run” tab. A Excel template file is generated to adjust the speed of the test as a function of the number of data points to be stored, as well as successive time intervals to store two successive images in the Python file. The start pane from LabVIEW GUI is clicked, followed by clicking on the “cycle start” pane in the Mach3Mill graphics user interface successively, and the initial voltage output from LabVIEW GUI is stored. LabVIEW is stopped after storing user-defined data points and further post-processing is carried out to calculate stress and strain. Based on the initial voltage output at the start of the test, initial pixel distance ‘Δl’, load cell calibration coefficient, cross-section area, stress, and strain are calculated. An Excel template is created to carry out this basic post-processing for the calculation of strain and stress.

## 7 Validation and characterization

Load Cell: The force acting on the load cell is calibrated by hanging different weights in the load cell and recording the corresponding voltage output. A special 3D printed base (Base Top) is employed to provide a firm base as the whole test setup is kept upside down during the calibration process. The two bases (Base Top and Base Bottom) are secured using a bracket (Load cell bracket) and M4 nut/bolt assembly. A special weight holder is 3D printed, and its weight is recorded. The holder is attached to the load cell using an M6 screw and corresponding voltage output from the LabVIEW is recorded. Multiple weights (from 0 to 1 kg) are hung simultaneously and corresponding voltages output are recorded. The output graph of the load cell is linear and the resulting slope of 0.702 ± 0.01 is used as the coefficient to calculate force. Similarly, calibration of the load cell, with a maximum load of 5 kg, for the planar tension test is carried out with a resulting slope of 6.8 ± 0.01.

Linear Actuator: Controlling lead screw-based actuator movement requires the number steps per unit of movement and its accuracy is depends on the lead screw characteristics. In our case, the lead screw had a nominal pitch of 5 mm and repeat positioning accuracy of ± 0.03 mm (manufacturer specification) and resolution of 0.001 mm (estimated based on the micro-steps). Mach3Mill module default setting for steps per unit is set at 2000 steps per unit (unit is 1 mm in our setting). This can be adjusted by entering user-defined values in the motor tuning tab. In order to calibrate the actuator travel distance and velocity, estimation of optimum steps per unit is essential to ensure distance in the Mach3 module results in identical physical travel distance. Firstly, we run the actuator with an input velocity of 1 mm/min and a travel distance of 10 mm starting from the default steps per unit setting of 2000 steps/unit. The input travel distance from the Mach3 Mill module and actual distance movement from the linear actuator is noted. This process is repeated until the input and output distances are equal (1300). Upon determination of optimum steps/unit value, the test is repeated (n = 7) to get repeatable output distance. The output graph was linear with $R^{2}=1$. Similarly, the time taken to travel the distance by the actuator is recorded to ensure the same input velocity during the test.

**Fig. 6:**
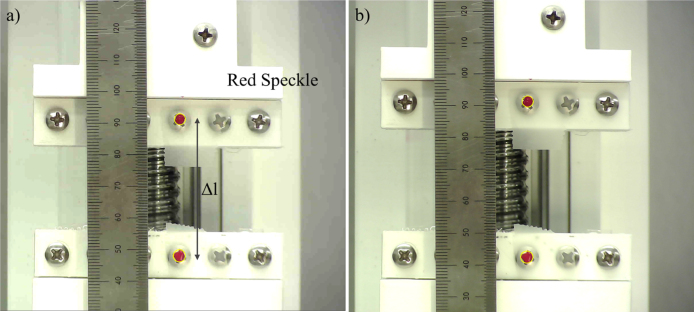
Calibration of DIC setup using a caliper, and images taken by Python.

DIC Camera: Strain evaluation is based on the measurement of the distance between two red dots in the DIC technique, which requires calibration (Fig. 6). For this purpose, the distance calculations based on the camera image, Python code, and a caliper are analyzed. An open-source computer vision library (OpenCV) is employed in Python for detecting red dots. Firstly, two red marks, representing red dots, and a ruler (Fig. 6) are attached to the gripper head of the test setup to calculate the actual distance based on the image. Next, the test setup is operated for a distance of up to 7 mm and images are recorded. Pixel units are used to measure distance. Here the pixel distance between two red dots in the image is calculated using MS Paint, and the pixel distance output calculated using the Python code is also recorded. Furthermore, the distance between two red marks from the ruler is also measured in the images. A caliper is also used for measurement of displacement in each step. The summary of strain calculation, using these three methods, is given in Table 1. The strain reported from Python and the caliper is reasonably close with a maximum observed difference of 0.008, which is significantly smaller than the error arising from the size of the red dot (discussed in the uncertainly section).

Uncertainty Estimation: In order to establish confidence in the result, the uncertainty in strain and stress was calculated using the error propagation principle. The variation in the capability of the DIC camera to recognize red dots depends on the diameter of the red dot. In addition, the red dot changes its shape from a circle to an oval shape with very large deformation, which leads to errors in estimating the center of the red circle. Therefore, the cumulative error in strains is mainly attributed to the error in the red dot diameter ‘d’, center to center distance between the red dots ‘l’, and initial length of the sample during the start of test ‘$\Delta l_{0}$’. Similarly, the error in the measurement of stresses arises due to the error in measurement of the thickness, the voltage’s output ‘V’ associated with force measurement, the width ‘w’, and the thickness ‘t’ associated with the cross-section for samples. The error bars for stresses and strains were calculated using Eqs. (6) & (7) followed by Eq. (8) for calculating uncertainty in elastic modulus.

(6) $$\Delta \sigma =\frac{\Delta V}{w\cdot t}+\frac{V\cdot \Delta w}{w^{2}\cdot t}+\frac{V\cdot \Delta t}{t^{2}\cdot w}$$

(7) $$\Delta ɛ=\frac{\Delta l}{l_{0}}+\frac{l\Delta l_{0}}{l_{0}^{2}}$$

(8) $$\Delta E=\frac{\Delta \sigma }{ɛ}+\frac{\sigma \Delta ɛ}{{ɛ}^{2}}$$

**Table 1:** Displacement calibration data.

S.No	Camera	Python	Caliper	Difference
	Strain	Strain	Strain	Strain

0	0.000	0.000	0.000	0
1	0.015	0.015	0.023	0.008
2	0.038	0.036	0.035	−0.002
3	0.053	0.056	0.058	0.002
4	0.073	0.075	0.070	−0.005
5	0.083	0.090	0.093	0.003
6	0.105	0.109	0.116	0.007
7	0.132	0.133	0.140	0.006
8	0.150	0.155	0.163	0.008
9	0.167	0.172	0.174	0.003

The uncertainty in the measurement of voltage ‘ΔV’ is taken as 1.2 mV according to the accuracy provided by the manufacturer. The uncertainty in the red dot diameter ‘Δd’ is assumed to be one-fourth of the diameter of the red dot. Considering the larger diameter of 2 mm in a worst-case scenario, Δd equal to 0.5 mm is adopted for the error bar calculation in strain. Similarly, uncertainties in the measurement of initial length ‘Δl’ originated due to compounding of inherent error in the caliper (± 5 microns) and error in measuring the distance between the center of red dots (0.5 mm). The uncertainty in the measurement of ‘$\Delta l_{0}$’ is estimated based on (a) the increment of strains recorded (b) the difference in the strain at zero stress level during the loading and unloading cycle, and (c) error in estimating center to center distance between red dots. The close proximity in strain level between two consecutive test data points and a small difference in strain level from both the loading and unloading curve lead to less error in estimating initial length ‘$\Delta l_{0}$’. Similarly, red dots with small diameters increase the accuracy in determining the center of the red dots. In our case, an error in the measurement of initial length due to the large diameter of red dots was dominant; therefore, the uncertainty in ‘$\Delta l_{0}$’ was chosen as one-fourth of the red dot diameter. The uncertainty in the measurement of the width ‘Δw’ is based upon the cumulative value of least count on the digital caliper, micrometer gauge (0.001 mm), and variation in width (0.05 mm). Variation in width is determined by measuring width at 5 different locations followed by statistical analysis (Student-t test). Similarly, uncertainty in the measurement of the thickness ‘Δt’ is based upon the collective value of the microscope’s calibration tool (± 10 microns) and statistical variation in thickness (0.002 mm). Variation in thickness is determined by measuring three thickness data at 3 different locations (each involves multiple measurements) followed by statistical analysis (Student-t test).

It is notable that the ultimate accuracy of the strain measurement could be improved with smaller speckles; however, this requires careful attention and is subject to limits. In a full HD camera, the Horizontal resolution is 1082 pixel. If a sample undergoes 100 percent stretch and if the speckles could be detected by 5 pixels, the strain resolution can become as small as 0.001. However, in practice here, we are limited to our ability to create small, uniform, and detectable speckles on transparent samples. Furthermore, upon large stretches, speckles can deform (from circle to oval shapes), which introduces another source of errors. Finally, extremely small speckles may represent the surface displacement as opposed to the whole sample. Therefore, while carefully applied speckles can improve the accuracy, we report an upper bound on the accuracy (in this example 0.01) that is estimated based on the diameter of speckles. In comparison, calculating strains from standard extensometer [60] can range from 0.1 (class A) to 1 (Class E) percent, or displacement verification from the actuator movement (and not the sample) classifies from 0.5 percent (Class A) to 3 percent (Class E) of the reading values [61]. As another example, a research test setup reported 1 percent error [15] on the strain that was measured from the actuator operation.

### 7.1 Sample preparation

**Fig. 7:**
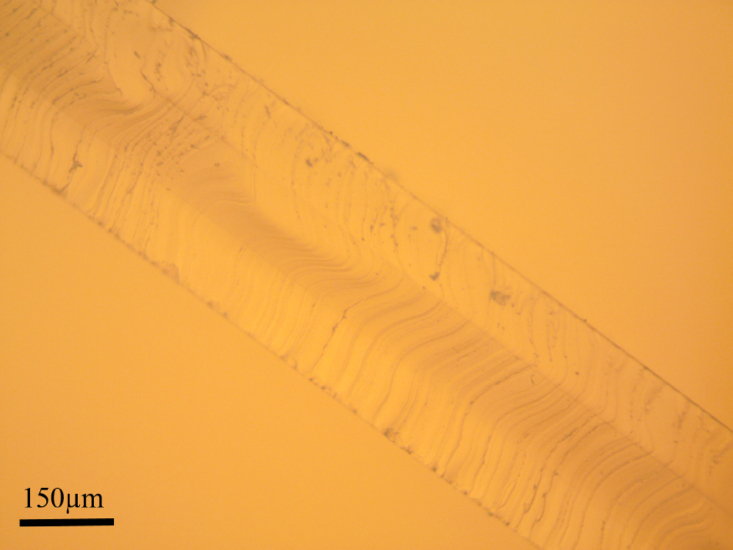
Sample cross-section image showing multiple layers of fabrication.

PDMS samples for the test are prepared from commercially available Sylgard 184® (Dow Corning, USA) in a 10:1 ratio, and mixed in a centrifugal mixer (AR-100 THINKY). The mixture is then further degassed to avoid bubbles trapped during the process of mixing. The PDMS is then spin-coated (Laurell Technologies Corporation, USA) at 500 rpm for 1 min to achieve each layer of thickness equal to 0.1 mm. Samples of varying thickness from 0.36 mm (Planar) to 0.38 mm (Uniaxial) were fabricated in multiple steps through spin coating (Fig. 7). The baking time for successive spin-coated layers was kept to 5 min at 100 °C on a hot plate (Microyn™) to avoid complete curing and proper bonding of PDMS layers. Upon spin coating of the last layer, the PDMS sample is left to fully cure in the hot plate (Microyn™) at 100 °C for an hour. The uniaxial test sample is then cut using a 3D printed die to yield test specimen shape as per ASTM D412 Type C. Here, the red dots (speckles) are placed on the two narrow ends of the sample that are at least 20 mm far from the grippers. Planar tensile tests require a high ratio of width to length of samples. therefore, a rectangular-shaped sample with 70 mm width and 30 mm length is cut. Both samples are then placed in an acetone bath for the lift-off process. The sample is lifted off (after staying it in the bath for 5 min) and washed with Isopropyl Alcohol. It is further further dried by blowing Nitrogen using an air gun. In order to remove erroneous results, the sample is optically characterized using a microscope (Amscope MU 503) to measure the thickness, whereas the width and length are measured using a caliper. Furthermore, two small dots having red color made from (Silc Pig™) are marked on the test section of the samples.

After mounting the planar tensile test sample, two red dots are painted on the sample, approximately 2–3 mm from the gripper. While increasing the distance from the gripper can reduce any undesirable effects of the grippers on the strain, it also reduces the cumulative deformation detected, thereby decreasing the strain measurement accuracy. Conversely, an excessive length of the sample may contribute to an increase in in-plane strain, deviating from the plane strain condition. We note that an optimal distance between the dots and the gripper could potentially improve the overall characterization technique. However, a detailed study on this matter is left for future work. In this work, instead, we focus on the repeatability of the measurements with the aforementioned technique and assess if the data is consistent with other works in the literature.

### 7.2 Tensile tests

**Fig. 8:**
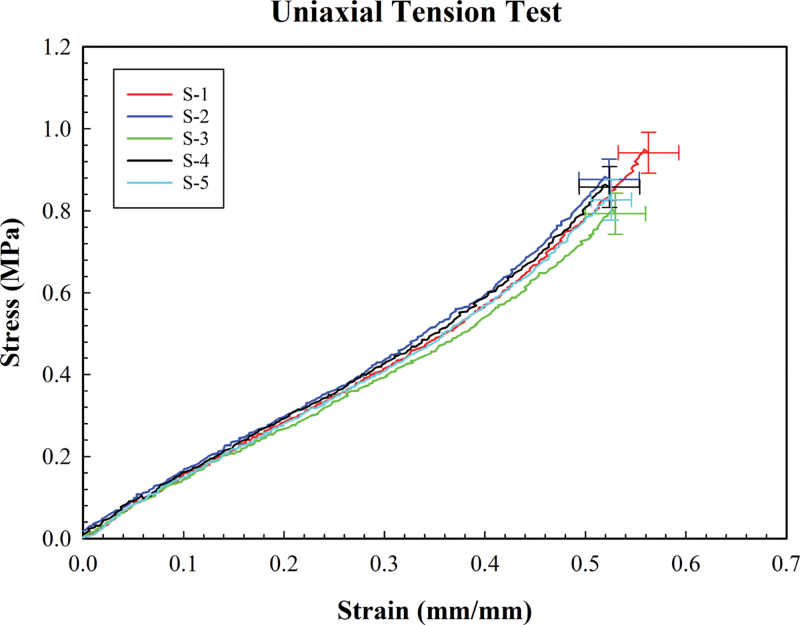
Uniaxial tensile test data.

**Fig. 9:**
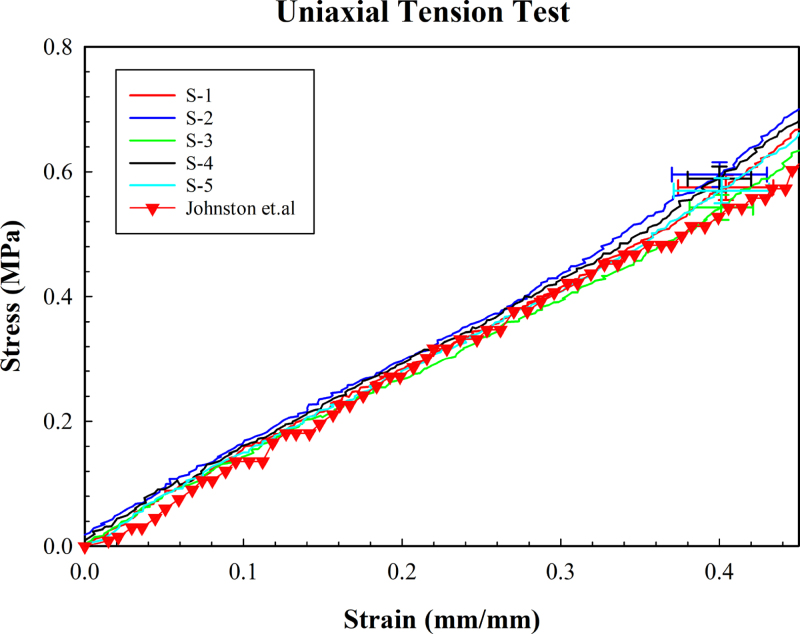
Uniaxial tensile test data and the data reported from other work.

The uniaxial tensile test was performed until the strain level of 50% to avoid distortion of red dots at higher strain levels (see Fig. 8). These test results, are also plotted against results from other reports (Fig. 9) . The test is executed through the LabView routine for one full cycle of loading and unloading. In order to correctly record the evolution of stress on the test sample, the test sample is slightly buckled and the test is executed to retrieve the force vs. displacement curve. The resulting force vs. displacement curve exhibited two lines where a flat line is observed at the start, followed by a linear line. The cut-off point for the calculation of elastic modulus is defined as the point where the flat line ends. The corresponding displacement at the cut-off point is used as a starting point for the calculation of stress vs. strain. The engineering stress vs. strain plot showed typical hyper-elastic behavior as shown in Fig. 8. We note that a sample when stretched at higher strains, can result in the camera stopping detecting the red dots. This could happen due to the fact that, at a certain stretch, the optical distortion prevents the Python program from reliably detecting the red dots. As a result, the maximum detectable strain varies among the samples.

**Fig. 10:**
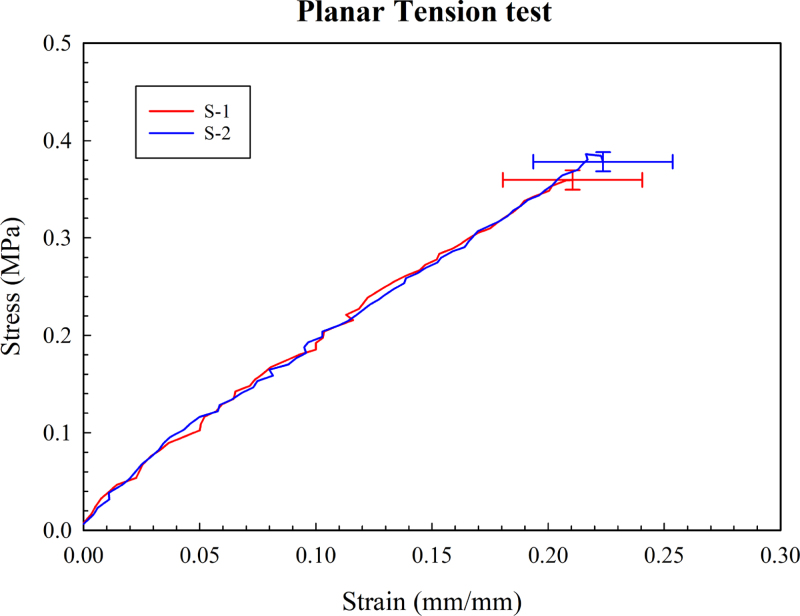
Planar tension test data.

Planar tensile tests were also carried out on PDMS samples that we fabricated by spin coating of PDMS and curing on a hot plate. Here, the tests (Fig. 10) were carried out from zero strain to that of 20%. This strain is lower than that of the uniaxial test because the sample in the planar tensile test typically ruptures at a lower strain. Here we carried out the test on two different samples with thicknesses of 0.36 mm. The stress–strain curves are reported in Fig. 10. Here, we notice that for the two tests and samples, we get nearly identical stress–strain curves, which grow our confidence in the repeatability of the results.

### 7.3 Hyperelastic coefficients’ assessment

**Fig. 11:**
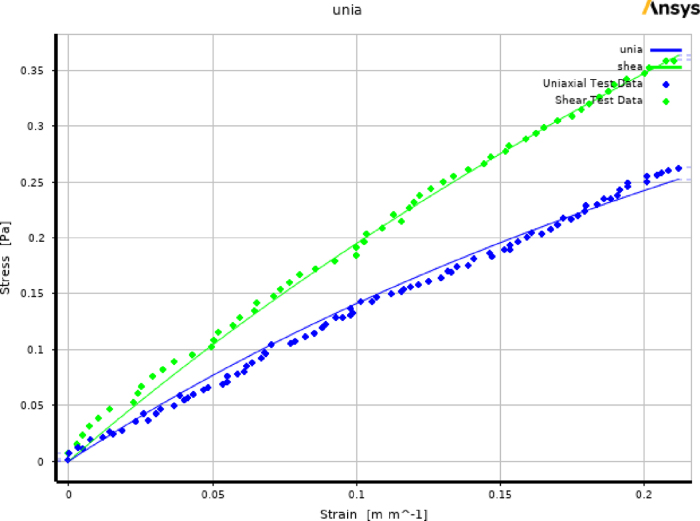
Curve fitting plot in ANSYS Workbench.

Mooney–Rivlin coefficients are evaluated by curve fitting various combinations of experimental data sets from uniaxial (Fig. 8) and planar tensile tests (Fig. 10) in ANSYS Workbench (Fig. 11). It is important to note that, in our experience, strain cut-off levels had to be identical for both uniaxial and planar tensile tests to avoid numerical instabilities. The strain cut-off point employed in this study was chosen at 20%. The data points were imported into the Engineering Data section of the Static Structural module in the workbench by creating new material. Once both data were provided, a two-parameter Mooney–Rivlin material model was selected and ANSYS provided the option for evaluating material properties $C_{1}\&C_{2}$ by curve fitting the data. The quality of curve fitting was governed by the choice made on “Error Norm for Fit” prior to solving for curve fit. Absolute error criteria were adopted during the curve fit process to minimize error during curve fit. A total of 10 different combinations of data resulting from 7 samples (5 uniaxial & 2 planar tests) were used in the evaluation of the hyperelastic material coefficients. Among 10 material constant data, 6 data sets had positive $C_{1}\&C_{2}$ values, whereas the remaining 4 data set had positive $C_{1}$ and negative $C_{2}$. The resulting coefficient value of $C_{1}$ varied from 385 to 185 kPa whereas the variation in $C_{2}$ was observed in the range of 97 to −103 kPa. The coefficient values on average are in the same order of magnitude close to the results obtained using the analytical approach ($C_{1}$ = 270 kPa and $C_{2}$ = 10.8 kPa) [52].

### 7.4 Validation of result

Uniaxial tensile tests for 5 PDMS samples showed a linear behavior until 40 percent strain (Fig. 9). This finding is consistent with an earlier report on the tensile test of uniaxial PDMS (Sylgard 184) on the linear relationship between the strain stresses [34]. Furthermore, this work showed that the stress at this strain varies from 0.5 Mpa (samples cured at 25 °C) to 0.8 MPa (samples cured at 100 °C in the oven). In our experiment, the stress was measured to be 0.5 Mpa. We note that our measurement of temperature on the hot plate showed a temperature of 70-80 °C rather than 100 °C. Therefore, our results are reasonably close to this report [34]. Here, samples with a thickness of 0.38 mm were used for the uniaxial tensile test. The samples were fabricated by spin-coating PDMS and baking on a hot plate. We note that inevitably the thicker samples will have a slightly lower average cure temperature up to 10 degrees. Moreover, the change in the thickness of samples results in slight differences in curing, which can affect the mechanical properties of PDMS. The resulting elastic modulus value varied from 1.29–1.41 MPa which is close to the reported value of 1.32 ± 0.07 MPa for samples cured at 25 °C [34]. A qualitative comparison of the difference in stress level at 40% strain is depicted in Fig. 9. In another instance, sample S-3 was subject to uniaxial tensile testing for 3 rounds to assess the repeatability of the result. The resulting elastic modulus value from the 3 test varied from 1.33–1.35 MPa. This showed the repeatability of the tests. We note that the mechanical behavior of each tested sample for strain levels up to 30% was nearly identical.

In order to establish further confidence in the results obtained from the curve fitting, we simulated the planar tests using the finite element analysis, in a Mechanical APDL environment. In the FEA, a two-parameter Mooney–Rivlin model was employed. The input parameters for the simulation were dimensions of the sample, $C_{1}\&C_{2}$, and displacement at 20% strain. Each material constant obtained from curve-fitting was then employed to validate the planar tension test.

**Fig. 12:**
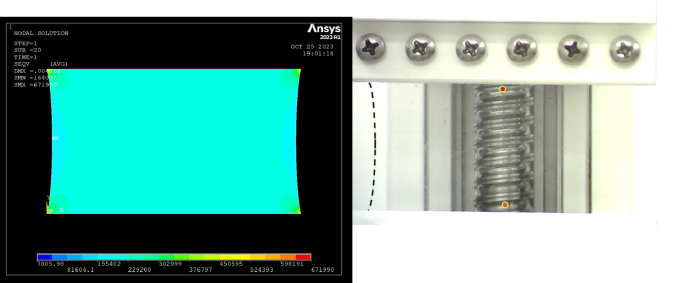
(Left)Von-mises stress distribution of planar tension test. (Right) Test sample deformation at 20% strain.

**Fig. 13:**
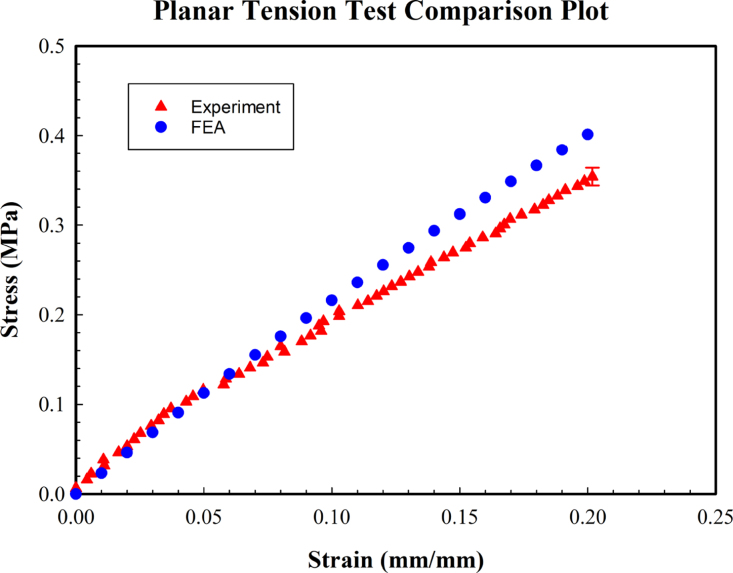
Planar tension test experiment Vs FEM result.

Planar shear tests were simulated using finite element analysis and using the coefficients curve-fit from the experiments. A two-dimensional quadrilateral structured mesh was used for simulating the planar tension test (Fig. 12). The lower end of the sample was constrained to move in X and Y directions, whereas the displacement, corresponding to a 0.2 strain, was applied at the top side. The convergence criteria for each step were set at 1E-8 for better results. The simulated shear test and the experiment are shown in (Figs. 12, 13), which are qualitatively in agreement. These simulations could also be use for quantitatively assessment of the curve-fit coefficients.

A total of 20 simulations were performed (for 10 sets of the constants found for two topologies corresponding to two planar tests). The simulations for each group (based on the two topologies) mimic the experiment performed, however, each simulation uses one of the hyperelastic constants pair reported in Table 2, Table 3. This comparison allows us to assess the largest error in simulation that results from the possible errors in the reported coefficients. One of these simulations is compared with the corresponding experiment in Fig. 13. Here, the resulting stress level from the simulation was consistent with the experiment. The maximum observed variation, among all comparisons made between the experiments and simulations, was found to be 28%. A difference in the stress level from simulation & experiment from 20 simulation results varied from 0.05 MPa to 0.1 MPa. A detailed comparison of the stress level for each material constant employed in finite element analysis is summarized in Tables 2 & 3. These comparisons further grow our confidence that the simulation and experiments are self-consistent and that the hyperlastic model, as well as the curve-fit coefficients were able to reproduce, the tensile test, reasonably accurately.

We note that the FEA results (Fig. 13) overestimate the experiment starting from a strain level of 8%. This overestimation can be due to a variety of reasons such as the difference in the location of the sample gripping position, inaccuracies in the curve fittings, or random or systematic errors in the reported values.

**Table 2:** Stress level comparison from FEA and Experiment for Planar Sample S-1.

$C_{1}$	$C_{2}$	ɛ (%)	$\sigma_{Exp}$ (MPa)	$\sigma_{FEA}$ (MPa)
385	−103	0.2	0.348	0.423
373	−89	0.2	0.348	0.425
333	−52	0.2	0.348	0.416
322	−39	0.2	0.348	0.417
274	6	0.2	0.348	0.408
264	18	0.2	0.348	0.409
240	40	0.2	0.348	0.404
229	52	0.2	0.348	0.404
196	84	0.2	0.348	0.398
185	97	0.2	0.348	0.40

We may further investigate the $C_{1}$ and $C_{2}$ in our experiment by assessing the resultant shear modulus from other work [52] and the values found here. Upon substituting curve fitted values of $C_{1}\&C_{2}$ into Eq. (4). The variation in shear modulus value obtained from curve fitting was < 5% from shear modulus value obtained using the coefficients found elsewhere ($C_{1}\&C_{2}$ [52]). Furthermore, the shear modulus value varied by less than 10% from values reported for the 10:1 mix ratio of PDMS [62]. Poisson’s ratio computed using Eq. (5) was in the range 0.48–0.54, including the error bar, while the expected value (0.4950 ± 0.001) for Sylgard 184 [63] is within this range.

**Table 3:** Stress level comparison from FEA and Experiment for Planar Sample S-2.

$C_{1}$	$C_{2}$	ɛ (%)	$\sigma_{Exp}$ (MPa)	$\sigma_{FEA}$ (MPa)
385	−103	0.2	0.354	0.453
373	−89	0.2	0.354	0.455
333	−52	0.2	0.354	0.445
322	−39	0.2	0.354	0.447
274	6	0.2	0.354	0.436
264	18	0.2	0.354	0.438
240	40	0.2	0.354	0.431
229	52	0.2	0.354	0.431
196	84	0.2	0.354	0.425
185	97	0.2	0.354	0.426

Although $C_{1}\&C_{2}$ values found using the FEM package were self-consistent such that all produced a shear modulus close to that of the literature and simulated the planar tensile tests were close the experiment (Fig. 13), the significant change in their values raises the question on why they vary largely. This observation is consistent with the fact that $C_{1}\&C_{2}$ are not unique if the test data is limited and fitting $C_{1}\&C_{2}$ with limited data is a delicate process. On the other hand, we observe that while the uniaxial and planar tensile test data points do not vary significantly (see Fig. 8, Fig. 9), the fit values for $C_{1}\&C_{2}$ significantly varied (in Table 2 from negative to positive values). Here, we note that the detailed choice of errors and method of curve fitting are not known for the results in Table 2, as we are using closed-source software. Various recent reports suggested that use of specific methods in curve fitting is crucial. Accordingly, we also curve fit the experimental data manually in a manner that is aligned with the recent literature. In our manual curve fitting (see Specification Table) we implemented the following points (1) we set a cut-off for the strains in the experimental data 0.2 (which makes the ultimate strain the same for both types of tests), (2) if the number of data points were different in the planar and uniaxial tensile test we multiply weights to the error for each type of test, that were inversely proportional to the number of data points, (3) we minimize the absolute error for ‘$\sigma /\left(\lambda^{2}-\frac{1}{\lambda }\right)$’ instead of ‘σ’ suggested by prior reports [6], [57], (4) we sample uniformly (equal spacing) from the stretches (‘λ’) in all regions.

**Fig. 14:**
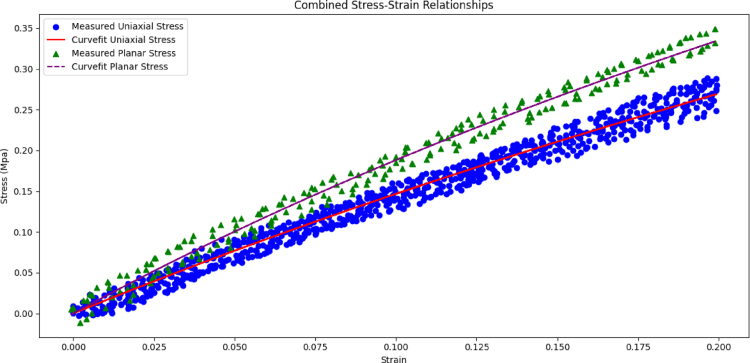
Manual curve fitting: that leveraged uniform sampling from the data, optimization of error from two types of test with equal weights that takes into account different number of datapoints, modified target function (i.e. error) in the optimization, and all datapoints from a total of 7 tests. The dots show the experimental datapoints for planar and uniaxial tensile tests and the lines show the curve fit values for the Mooney–Rivlin’s model. The program is provided in Specification Table.

The manual curve fitting with above mentioned assumptions is provided in Fig. 14 that was applied to all data points . Here, all 5 uniaxial tensile loading are depicted in blue circles, and two measurements for planar tensile tests are depicted in green triangles. The curve fit theoretical lines are also depicted in red and purple lines. It’s resulting $C_{1}\&C_{2}$ become 251 kPa and 18 kPa respectively, that are close to those reported elsewhere [52] ($C_{1}$ = 270 kPa & $C_{2}$ = 10 kPa). Here, we note that the two Mooney–Rivlin coefficients and shear modulus that were curve fit were no more than 20 kPa far from those reported values. This agreement further grows our confidence that a meticulous curve-fitting methodology along with increased number of data points could improve the accuracy of the hyperelastic coefficients. We further note that the choice of error (normalized or absolute), uniform sampling from various regions, or transformation of the error ($\sigma /\left(\lambda^{2}-\frac{1}{\lambda }\right)$ vs. σ) were found to be influential in the curve-fitting process (see Fig. 14).

In summary, while the variation in the range of for the curve-fit values for $C_{1}$ and $C_{2}$ are large (Table 2, Table 3), the resultant shear modulus and Young’s Modulus are both in agreement with those found in the literature. Furthermore, the experimental stress–strain curvatures (Figs. 8, 9) were repeatable and close to those found in elsewhere (Fig. 8). Additionally, the simulations using finite element analysis, with the curve-fit values for $C_{1}$ and $C_{2}$, were self consistent with the experiment. Based on these findings we grow our confidence that the experimental stress–strain curves (Fig. 8, Fig. 9) are valid. However we also note the change in the curve-fit value $C_{1}$ and $C_{2}$ may arise from the fact that numerical curve-fitting is challenging as indicated in the prior reports [6]. Another more likely explanation, given that the simulation is in good agreement with the experiment, is that the fact that $C_{1}$ and $C_{2}$ are not unique and different values may result in the same simulation [64], specially if the combination of tests/loads are limited. Common strategies to overcome the challenge in curve fitting are increasing the number of data points/tests, optimizing the error in terms of $\sigma /\left(\lambda^{2}-\frac{1}{\lambda }\right)$. We found that, after implementing these points in a manual curve fitting process, the curve-fit coefficients are closer to those reported in the literature. Accordingly, while the results suggest the utility of the proposed method in assessment of the Mooney Rivilin’s coefficients, it also suggests the need for meticulous curve fitting, and/or validation of the curve-fit values for the simulation/modeling complex problems.

## 8 Conclusion

Here, we report a combination of computational and experimental pathways tailored for the characterization of the hyperelastic material PDMS by developing a low-cost 3D-printed uniaxial and planar tensile test setup. The setup is ideal for the characterization of thin film samples. The small form factor is especially suitable for characterizing materials to be used in medical devices. This cost-effective method employs open-source packages, off-the-shelf components for uniaxial and planar tensile tests, and 3D-printed parts. A low-cost method was developed for logging the data using Python and LabVIEW to calculate the strain. Two-point red circles were successfully employed and validated for strain calculation during the characterization process. Validation of data involved the characterization of PDMS samples with sub-mm thickness varying from 0.36 mm to 0.38 mm, ideal for small form factor medical devices. Uniaxial tensile tests confirmed qualitative hyperelastic behavior of PDMS, as a nonlinear regime was observed for strain greater than 40%. Furthermore, the tensile tests carried out were repeatable within the margin of accuracy we calculated for five measurements of five different samples, which was very close to those reported elsewhere [34]. The Young’s modulus resulting from the uniaxial tensile test was also found to be less than 5% different from other work [34]. We also carried out planar tension tests for two samples with their strains limited to 20%. The experimental data from two types of tests were used to find two-parameter Mooney–Rivlin coefficients. We observed significant variability in the values of coefficients when compared with other work [52]. However, the shear modulus resulting from the experimental data was reasonably close to those extracted from other reports (less than 5% percent from [52] and less than 10% from [62]). When we implemented a meticulous curve-fitting approach, including definition of error as advised in the literature, we observed that $C_{1}$ and $C_{2}$ are significantly closer to the reported values in the literature (270 vs. 251 kPa and 10 vs. 18 kPa ). In summary, the methodologies demonstrate the potential repeatability and accuracy needed for the simulation of practical engineering problems involving hyperelastic materials; however, similar to prior reports meticulous curve-fitting and additional types and number of tests are needed, when complex models with complex loading conditions are involved.

Although we observed great agreement with a significant number of reports, in terms of Young’s and Shear moduli, and the hyperelastic coefficients demonstrating the potential for the proposed methodology, there are certain aspects of this work that need to be improved in the future. Improvements can include the addition of biaxial tensile tests, uniaxial compression tests, pure shear tests, and volumetric tests. Studies on more samples and data points, samples made from a variety of materials, sample dimensions, process parameters for the samples, or other curve-fitting techniques are among the efforts envisioned to understand the limitations of the proposed technique. Another potential area could be validating the simulation for specific applications using the characterization data achieved by the proposed tools. Such tests can assist in the estimation of higher-order Mooney–Rivlin coefficients and can be carried out using the proposed process to characterize large strain behavior. Finally, since some parts of the tools are mostly made from 3D printed parts, the addition of environmental containers (controlling aqueous media and temperature) can be highly beneficial for researchers applying elastomers in the area of medical devices, implantable devices, and medicine.

## CRediT authorship contribution statement

**Hemanta Dulal:** Writing – original draft, Visualization, Validation, Investigation, Formal analysis, Data curation. **Seyedhamidreza Alaie:** Writing – review & editing, Supervision, Software, Design, Visualization, Validation, Investigation, Resources, Project administration, Methodology, Funding acquisition, Conceptualization.

## Ethics statements

The authors declare that no human being or animal were used in this work.

## Declaration of competing interest

The authors declare no any competing interest to their best knowledge.
